# Dialogue with the Giants of Microsurgery: Professor Fu-Chan Wei and Professor Joon Pio Hong

**DOI:** 10.1055/a-2113-3364

**Published:** 2023-10-05

**Authors:** Ankur Khajuria, Wei F. Chen, Jung Ju Huang, Susana Heredero, Joon-Pio Hong, Fu-Chan Wei, Tommy Nai-Jen Chang

**Affiliations:** 1 Kellogg College, University of Oxford, Oxford, United Kingdom; 2 Department of Surgery and Cancer, Imperial College London, London, United Kingdom; 3 Department of Plastic Surgery, Cleveland Clinic, Cleveland, Ohio; 4 Division of Reconstructive Microsurgery, Department of Plastic and Reconstructive Surgery, Chang Gung Memorial Hospital, Linkou Medical Center; Chang Gung Medical College and Chang Gung University, Taoyuan, Taiwan; 5 UGC de Cirugía Maxilofacial, Hospital Universitario Reina Sofía, Córdoba, Spain; 6 Department of Plastic Surgery, Asan Medical Centre, Seoul, Korea; 7 Founder, International Microsurgery Club, Taiwan

The International Microsurgery Club (IMC) was created by Dr. Tommy Chang (Taiwan), with the webinar series filling the coronavirus disease 2019 (COVID-19)-induced knowledge gap and providing a platform for microsurgery masters to share knowledge. ^1^ As of April 2023, IMC had more than 19,200 members worldwide. ^2^ An IMC poll identified Professors Fu-Chan Wei and Joon-Pio Hong as the most influential teachers in microsurgery. This article summarizes the lessons from the “Dialogue with the Most Influential Teachers in Microsurgery” webinar on April 9, 2023, divided into technical, nontechnical, and life lesson sections.

## Technical Aspects of Microsurgery

Q: Any tips or tricks to reduce venous congestion in superficial circumflex iliac artery perforator (SCIP) flap?

A: Prof. Hong explained that initially he primarily used the medial perforator of the SCIP flap but faced issues with venous congestion and partial flap loss. By including the superficial vein within the middle of the axis of the flap design helped overcome these problems. With ultrasound guidance, he can now precisely locate and incorporate the vein, reducing the risk of complications.

Q: What is the optimal timing of reconstruction in the diabetic foot after vascular intervention?

A: Prof. Hong advised prompt reconstruction after angioplasty or revascularization in the diabetic foot, ideally within 1 to 2 days. Reocclusion rates can be high (60% in 2 months) and the ischemic foot is susceptible to inflammation, infection, and other complications. Seizing the opportunity while the vessel is open is critical to avoid further issues.

Q: What is the utility of a duplex ultrasound in perforator flap surgery?

A: Prof. Hong utilizes ultrasound to locate perforators and assess flow velocity, which provides both anatomical and physiological information. The pathway and recipient source can be determined using flow volume, and dissection effort and muscular course are also taken into consideration. Prof. Hong suggested reading papers, watching tutorials, and borrowing a duplex ultrasound machine to understand Doppler. A 10- to 20-MHz probe is adequate for perforator mapping. Ultrasound has significantly impacted Prof. Hong's practice.

Q: What are your views regarding the concept of the “reconstructive elevator”?

A: Prof. Hong and Prof. Wei agreed that while the reconstructive ladder had its place in the history of microsurgery, the reconstructive elevator is a more patient-centered approach that allows for the use of the most advanced techniques to provide the best results for patients, through a more tailored and individualized approach to reconstruction.

Q: For anterolateral thigh (ALT) flap dissection, is it better to start from the perforator to the pedicle or vice versa?

A: Prof. Wei suggests starting from the pedicle, working from distal to proximal, tracing the vessels from the superficial plane and gradually dissecting toward the source artery.

Q: Does drawing help to artistically perform microsurgery?

A: Both Prof. Wei and Prof. Hong believe that while drawing is not necessarily important, record-keeping for preoperative, postoperative, and surgical records is critical. Prof. Wei highlighted the importance of picture-taking, particularly for complex procedures like head and neck reconstruction, and encourages the organization of records through digitization and computer skills training.

Q: Are there any tips or tricks to avoid kinking in the blood vessels if the pedicle is high up and the perforator descends for hemimandibulectomy reconstruction using free fibula?

A: Prof. Fu Chan Wei suggests turning the flap around to reach the opposite side of the neck to avoid kinking.

Q: Can one perform aesthetic surgery, after a career in microsurgery?

A: Prof. Hong emphasizes the interconnectedness of reconstructive and aesthetic surgery. He thinks that reconstruction without an aesthetic component is incomplete and that microsurgeons should aim for both aesthetic and functional outcomes.

## Nontechnical Aspects of Microsurgery

Q: How does one build a successful microsurgery team?

A: Prof. Wei suggests starting with a small group of like-minded individuals, specializing in a particular field, and gradually expanding the team through accumulated experience. He emphasized the importance of hospital and national health care system support in developing a strong team, sharing his experience of building a team of 18 to 19 individuals over time. Ultimately, the success of a microsurgery team depends on a strong leader, supportive hospital system, and dedicated team with shared goals and specialties.

Q: How to establish microsurgery services in developing countries?

A: Prof. Wei suggests starting with simple cases and accumulating experience to gain hospital administration support for microsurgery growth. Prof. Hong notes ways to provide reconstruction even with limited resources, such as using local flaps or borrowing tools from other departments. He said that even with 3.5× to 4× magnification loupes, one can do microsurgery in large vessels if a microscope is not available and that some surgeons from developing countries have used cosmetic tweezers as forceps for microsurgery.

Q: How should COVID-19 and new technologies change the organization of plastic surgery residency programs in 2023?

A: Prof. Hong acknowledged virtual meetings' accessibility and knowledge-gathering benefits but stressed the significance of hands-on experience for becoming a skilled surgeon. He also emphasized understanding residents' long-term goals to align them with departmental needs. Prof. Wei emphasized developing relationships and fellowships to learn microsurgery, favoring hands-on practice over virtual or animal learning.

Q: Professor Eric Santamaria from Mexico asked “How to train residents in microsurgery when there aren't enough places in the world to offer fellowship training?”

A: Prof. Santamaria suggested that residents should learn to perform 4 to 5 free flaps after their plastic surgery residency, without needing a formal fellowship, to increase the number of competent microsurgeons in countries without enough training programs. Prof. Hong acknowledges the need for micro-fellowships, and notes that many residents lack exposure to microsurgery during their residency. He believes fellowships offer a crucial opportunity to gain this experience and that success depends on the candidate's interest and exposure to the practice.

## Life Lessons from the Master Surgeons

Q: Should one specialize in multiple flaps, or focus on a few and master them?

A: Prof. Wei reflected on his own experience, noting that when he was younger he tried to learn everything he could and would come back from conferences eager to try new techniques. However, over time he realized that with limited time and energy, it is not possible to pursue and master every technique. He suggested that young surgeons should focus on a few flaps, as pursuing too many will leave them as followers rather than leaders in the field. He recommended identifying and mastering a few flaps for soft tissue and bone reconstruction that can solve most problems. Prof. Wei himself has focused on the ALT and fibula flaps, which have been sufficient for majority of his cases.

Q: How do you deal with the stress and frustration of the work?

A: Prof. Wei suggests that working in a group where each member specializes in a particular area can help alleviate stress and frustration. He explained that his department is divided into four groups: trauma-related surgery, head and neck surgery, breast and lymphoedema surgery, and peripheral nerve and muscle functioning surgery. He emphasizes the importance of accumulating experience, talking to others, and reflecting on oneself to improve the results of patient care. Overall, Prof. Wei's advice focuses on specialization and collaboration to improve the quality of microsurgery.

Q: Do you remember the most challenging case you faced as a microsurgeon?

A: Prof. Hong recounted a case in his fellowship year where he was so eager to perform a free flap on a patient with an exposed tendon on the foot that he debrided extensively and put a free flap on it. His professor then told him that the patient could have just had a clean debridement or secondary intention healing, and that he should have considered the patient's perspective before performing the flap. This taught him the importance of putting the patient's needs first and considering if he would want a free flap if he were in the same situation. Prof. Wei emphasized the importance of seeking second opinions and reading articles before making decisions, especially for young microsurgeons, who may not have enough experience. He also emphasized the importance of applying microsurgery at the right time and in the right situation to achieve successful outcomes.

Q: How to overcome disappointment after a flap failure and how to prepare for the next flap?

A: Both Prof. Hong and Prof. Wei agreed that failure is inevitable. The key is to learn from it and understand why it happened. It is important to educate patients on the possibility of failure, as it can occur for the most skilled microsurgeons. In case of consecutive failures, taking a break, talking to mentors and colleagues, or taking a vacation are recommended. Additionally, Prof. Hong jokingly suggests drinking whiskey to cope.

Q: How to maintain balance between work, family, and health while practicing microsurgery?

A: Prof. Wei believes that balancing family life and a professional career in microsurgery is challenging. Sacrifices may need to be made for success, but he suggests spending as much time as possible with family when not working and including them in social activities. Trainees should pursue microsurgery with dedication and accept the sacrifices that come with it.

Q: How does once achieve the balance between innovation and inherent risks in developing new surgical procedures or flaps?

A: Both Prof. Hong and Prof. Wei agreed that innovation inherently follows risk and that surgeons must excel at current techniques before attempting new ones. Prof. Hong emphasized that innovation is about making small tweaks that lead to evolution. Young surgeons should write down their new ideas and revisit them once they have the necessary experience. Prof. Wei added that not everyone is qualified to innovate and stressed the importance of adequate knowledge and literature review before attempting new techniques. Completely new ideas must be validated through animal studies and peer review before proceeding. The importance of mentorship and literature review was emphasized.

Q: As a young microsurgeon, how does one choose a subspecialty?

A: Prof. Hong suggested that young plastic surgeons should ask themselves what they enjoy most about plastic surgery and what they are good at to help identify their field of interest. Focusing on a specific field allows for deeper expertise and the opportunity to contribute new ideas. Prof. Hong got into diabetic foot reconstruction as he identified it as an underserved field in plastic surgery during his senior residency. He encourages surgeons to identify underserved fields in their area and immerse themselves in a large patient pool to build experience and help patients in need.

Q: Is publication important in microsurgery?

A: Prof. Hong advised that publishing papers is important to communicate and contribute to the field. As one's writing progresses, the focus should shift from asking questions to answering the questions and challenging the status quo. Curiosity often leads to good research.

Q: What keeps you motivated?

A: Prof. Hong feels a sense of calling to give back and help others grow, which motivates him. He also discussed how the many breakthroughs and innovations, for example, perforator flaps, supermicrosurgery, and robotics, keep him excited. Becoming an expert and being open to new ideas/challenges is important for personal and professional growth. Prof. Wei is motivated by his passion for helping patients and the intellectual challenge of working on complex cases. He explained that his career as a microsurgeon was completely unexpected, and he became interested in microsurgery during his fellowship in Toronto, where he met his mentors. He was in the early stage of microsurgical development and was able to participate in new developments including toe-to-thumb transfer, osteocutaneous free fibula flap, and with Prof. Koshima, development of the perforator flap.

Q: How can early career surgeons balance their own skill development with teaching and training future generations?

A: Prof. Hong stresses the importance of critical thinking and developing one's own algorithm and logic in surgical processes. He mentioned that technical skills are just a small portion of surgical training, and that the ability to think critically about patient care is the most important aspect. He also emphasizes the importance of hands-on experience and having a clear understanding of the critical steps in surgical procedures before allowing trainees to perform them on their own. Prof. Hong believes that there is no alternative way to improve surgical skills without being hands-on.
